# Polypharmacology of small molecules targeting the ubiquitin–proteasome and ubiquitin-like systems

**DOI:** 10.18632/oncotarget.3917

**Published:** 2015-04-23

**Authors:** Ivano Amelio, Vivien Landré, Richard A. Knight, Andrey Lisitsa, Gerry Melino, Alexey V. Antonov

**Affiliations:** ^1^ Medical Research Council Toxicology Unit, Leicester, UK; ^2^ Institute of Biomedical Chemistry of The Russian Academy of Medical Sciences, Moscow, Russia; ^3^ Department of Experimental Medicine & Surgery, University of Rome “Tor Vergata”, Rome, Italy

**Keywords:** UPS, UBL, SUMO, UBC13, UCH37

## Abstract

Targeting the ubiquitin–proteasome system (UPS) and ubiquitin-like signalling systems (UBL) has been considered a promising therapeutic strategy to treat cancer, neurodegenerative and immunological disorders. There have been multiple efforts recently to identify novel compounds that efficiently modulate the activities of different disease-specific components of the UPS-UBL. However, it is evident that polypharmacology (the ability to affect multiple independent protein targets) is a basic property of small molecules and even highly potent molecules would have a number of “off target” effects. Here we have explored publicly available high-throughput screening data covering a wide spectrum of currently accepted drug targets in order to understand polypharmacology of small molecules targeting different components of the UPS-UBL. We have demonstrated that molecules targeting a given UPS-UBL protein also have high odds to target a given off target spectrum. Moreover, the off target spectrum differs significantly between different components of UPS-UBL. This information can be utilized further in drug discovery efforts, to improve drug efficiency and to reduce the risk of potential side effects of the prospective drugs designed to target specific UPS-UBL components.

## INTRODUCTION

The ubiquitin–proteasome system (UPS) is essential for the turnover and biological function of most proteins [1-3]. In short, ubiquitin is a protein with 76 amino acids, which is activated in an ATP-dependent manner by a ubiquitin-activating enzyme commonly referred to as E1. In the next step ubiquitin is transferred by an ubiquitin-conjugating enzyme (commonly referred to as E2) that, together with an ubiquitin-protein ligase (commonly referred to as E3), specifically attaches ubiquitin to a target protein through the amino group of a lysine residue [4]. The targeted protein can be polyubiquitylated (the addition of several ubiquitins to a single lysine residue in a protein) or multiubiquitylated (addition of single or several ubiquitin(s) to several lysines in one protein). Initially, protein ubiquitylation was considered only in the context of signalling for 26S proteasome-mediated degradation of the targeted protein (the case of polyubiquitination) [5]. Our current understanding, however, is much wider: there are diverse, nondegradative functions of protein ubiquitylation including the regulation of DNA repair,transcription, mRNA metabolism and splicing [1, 6-10]. Moreover, the 26S proteasome is subject to ubiquitination by itself, which affects its activity [11]. In addition, protein ubiquitylation is regulated tightly by complex mechanisms and is a reversible process: ubiquitin molecules can be removed by deubiquitylating enzymes (DUBs) [12].

Recently a number of regulatory cascades very similar to UPS have been discovered, the ubiquitin like modifiers (UBL) [13, 14]. By marking proteins with ubiquitin-like conjugates, the cell regulates activity of multiple downstream proteins and, thus, controls many important regulatory pathways. The structure of enzymological reactions that are involved in protein modifications by ubiquitylation is analogous to that used by ubiquitin-like cascades. One of the best studied ubiquitin-like proteins is small ubiquitin-related modifiers SUMO [15]. The conjugation of SUMO to target proteins regulates cell-cycle control, nuclear transport and the response to viral infections [16]. Three SUMO isoforms are currently known in man with functionally distinct roles. At present, about a dozen different ubiquitin-like cascades have been described with varying degrees of completeness [17].

Destabilization of the normal UPS function as well as defects in functionality of UBL signalling cascades have been shown to be linked to multiple pathological conditions such as cancer and neurodegenerative disorders [17, 18]. Although our understanding of the role of the UPS-UBL in various complex diseases is far from complete, there are examples of successful drug developments targeting the system. Bortezomib is the first drug targeting the UPS (inhibition of the proteasome) that was approved by the FDA for treatment of multiple myeloma in 2003 [19]. The selectivity of proteasome inhibition for killing tumour versus normal cells (normal cells also experience some toxic effects due to abnormal function of the proteasome) by Bortezomib was somewhat surprising. The commonly accepted explanation for this is that tumour cells (in particular white blood cells normally responsible for producing antibodies) have higher concentrations of aberrant proteins that are constantly degraded by the UPS making them more sensitive to the effects of proteasome inhibition [17, 18].

The success of Bortezomib along with our increasing understanding of the UPS-UBL and its potential role in human diseases has provoked significant interest in the development of drugs that can target specific components of the UPS-UBL pathways, thus modifying UPS-UBL function [18]. The current trend is the development of compounds targeting specific proteins of the UPS [20]. There have been multiple efforts recently to develop high-throughput screening (HTS) assays to identify drugs that modulate the activity of different components of ubiquitin or ubiquitin-like cascades [21-23]. In the public domain, we were able to find more than a dozen of such HTS (see Table 1), with each reporting thousands of molecules to be efficient modulators and, therefore, each reporting thousands of potential drug leads to act on specific UPS or UBL components.

The current process of drug discovery and subsequent (pre-)clinical development involves several stages; identification of molecules that act on a specific target is only the first step in this timely process. In the next step selected drug candidates are subjected to various tests to investigate potential off targets which would predefine their specific toxicity or side effects [24]. On the one hand, selection of many drug candidates obviously increases the chances to find the one with optimized safety parameters; on the other hand, testing many drug candidates towards a whole spectrum of potential off targets is virtually impossible. At this stage, it is imperative to introduce some rationale for selecting potential risk factors (such as defining the most probable off target effects) and, therefore, to optimize the number of tests needed. This rationale could be introduced by mining of publicly available HTS screens covering a wide spectrum of potential drug targets in various complex diseases.

In this article we are going to review several recently published high-throughput screening assays that aimed to identify potential drug leads targeting different components of the UPS-UBL (see Table 1). Furthermore, we provide comparative analyses of these screens versus the large number (> 1000) of HTS target-oriented screens publicly available in the PubChem repository. The possibility of such comparison arises from the lucky coincidence that the chemical libraries used in most of these screens are very similar, i.e. screens done independently by different labs with biologically independent assays (targets) share a significant number of the same screened molecules. This allows one to compute for UPS (UBL) targets the cross target correlation versus the spectrum of other targets available in PubChem: the odds of molecules to target any protein from the spectrum if the molecule targets the UPS (UBL). Therefore, for each UPS (UBL) target we obtain a list of top off targets which have high odds to be targeted by potential drug leads. We also provided analyses of discovered top potential off targets on the subject of drug efficiency and drug safety issues.

**Table 1:** High-throughput screening assays publicly available in the PubChem database modelling different components of ubiquitin or ubiquitin-like cascades

Assay ID	Assay Title	Active Molecules	Screened Molecules
485273	uHTS identification of UBC13 Polyubiquitin Inhibitors via a TR-FRET Assay	1540	328 071
588478	A screen for small molecule inhibitors of the human deubiquitinating enzyme, UCH37	1078	329 843
602429	uHTS identification of SUMO1-mediated protein-protein interactions	1206	362 962
2716	Luminescence Microorganism Primary HTS to Identify Inhibitors of the SUMOylation Pathway Using a Temperature Sensitive Growth Reversal Mutant Mot1-301	3324	315 446
2006	uHTS HTRF assay for identification of inhibitors of SUMOylation	1039	289 855
2540	HTS Luminescent assay for identification of inhibitors of Sentrin-specific protease 8 (SENP8)	4122	326 358
2599	uHTS Luminescent assay for identification of inhibitors of Sentrin-specific protease 6 (SENP6)	5820	324 660
434973	uHTS Luminescent assay for identification of inhibitors of Sentrin-specific protease 7 (SENP7)	4906	326 853
602440	uHTS Fluorescent Assay Using Nedd8 Protein Substrate for Identification of Inhibitors of Sentrin-Specific Protease 8 (SENP8)	2342	361 826
624204	uHTS identification of small molecule inhibitors of the catalytic domain of the SUMO protease, SENP1 in a FRET assay	774	363 394

### Inference of off target spectrum for UPS (UBL) targets

PubChem BioAssay [25] repository stores the results of biochemical HTS target oriented assays. Based on the PubChem BioAssay data model, results of HTS target oriented assays, in simple terms, could be interpreted as a subset of small molecules reported to inhibit (in rare cases activate) the targeted protein. The abundance of this information (> 1000 independent assays) from the PubChem BioAssay repository could be integrated on a large scale to derive binding spectra for approximately half of the million molecules across several hundred proteins. Many of these molecules have been tested in UPS (UBL) assays. Let us denote KA to be the number of active molecules (molecules exhibiting activity in the assay at relevant concentrations) in the HTS UPS (UBL) assay and as KB the number of inactive molecules. For each off target protein “Z” we count the number (*kA*) of the active molecules (from the UPS assay) which target “Z” (have been reported in the other HTS assay as active versus “Z”) and *kB* is the number of inactive molecules which target Z. Odds for the molecules (to be active) targeting “Z” is (*kA/kB*) while the same odds for the molecules which do not target “Z” is ((*KA-kA*)/(KB-kB)). The odds ratio is (*kA/kB*) / ((*KA-kA*)/(*KB-kB)*) and indicates the increase/decrease of odds for a molecule to target “Z” while targeting the UPS-UBL protein. Significance of the odds ratio is computed using *X*^2^- distribution and the adjustment of p-values for multiple testing (each target one hypothesis) is done using the FDR procedure [26-29].

### UBC13 polyubiquitin inhibitors

Ubc13 is an unusual E2 ubiquitin-conjugating enzyme which is only active as a heterodimer with another E2 enzyme and produces polyubiquitin chains exclusively linked at lysine residue 63 of ubiquitin. Ubc13 specifically ubiquitinates Tumor Necrosis Factor Receptor-Associated Factors (TRAFs), a family of adapter proteins. Ubc13-mediated ubiquitination of TRAFs is recognized as a critical step in signalling by TNFRs during the innate and acquired immune responses [30]. Inhibition of Ubc13 is considered as a potential strategy for development of novel immunosuppressive, anti-inflammatory agents as well as agents for treatment of neurodegenerative diseases [31]. *In vitro* high-throughput screening assays based on the principal of time-resolved fluorescence resonance energy transfer (TR-FRET) have been developed to identify potential inhibitors of Ubc13 activity [32]. Terbium-ubiquitin and fluorescein-ubiquitin have been used to generate a FRET reaction. In total, a library of 328 071 compounds were screened and 1540 were reported as active (efficiently inhibiting Ubc13 at clinically relevant concentrations).

The top ten potential off target activities for the molecules inhibiting Ubc13 are presented in Table 2. for example, the ability of a molecule to inhibit Ubc13 significantly increases the odds (~ 40 fold) for the molecule to additionally inhibit APAF1. In total, 2314 (406 + 1908) molecules which were tested in the Ubc13 screen demonstrated the potential to inhibit APAF1 and 406 of them exhibited potent inhibition of Ubc13 while 1908 did not. Observed odds for a molecule to inhibit Ubc13 in the screen is ~ 0.006 (1538/223822), while observed odds for the molecules experimentally validated to inhibit APAF1 is approximately 40 times higher ~ 0.21 (406/1908). Thus, we observe a strong association between Ubc13 and APAF1: molecules targeting one protein have a reasonable chance to target the other as an off target.

Recent evidence has implicated the E3 ligase activity of TRAFs in the pathogenic aggregation of mutant proteins in neurodegenerative diseases such as Huntington disease [33]. Instead of conventional polyubiquitination, TRAF6 promotes atypical ubiquitination (with the Ubc13 as the E2) of mutated misfolded proteins and, thereby, prevents them from degradation [34, 35]. Recently APAF1 dominant negative inhibition was tested for its anti-apoptotic effect on degenerating nigrostriatal neurons in a 1-methyl-4-phenyl-1,2,3,6-tetrahydropyridine (MPTP) model of Parkinson's disease and was shown to inhibit MPTP toxicity [36]. Thus, both Ubc13 and APAF1 are potential therapeutic targets in Parkinson's disease but with different mechanism of action. Our analysis reveals that Ubc13 and APAF1 are frequently targeted together by small molecules as observed from the available HTS screens. This makes the Ubc13 and APAF1 pair an attractive multitarget for development of therapeutics with polypharmacological mechanisms of action in Parkinson's disease.

**Table 2:** Top off targets for molecules inhibiting UBC13 (based on data from assay “uHTS identification of UBC13 Polyubiquitin Inhibitors via a TR-FRET Assay”)

(Target): activity	Odds Ratio	kA*	*KA* (The Number of Active molecules)	kB**	KB*** (The Number of InActive molecules)	*P*-value
(MAP4K2):inhibitor:mutant	53.06	231	1538	743	223822	< 1.57E-280
(APAF1):inhibitor	41.71	406	1538	1908	223822	< 1.57E-280
(RAD52):inhibitor	36.35	140	1538	615	223822	1.24E-152
(MAP4K2):inhibitor	27.8	395	1538	2748	223822	< 1.57E-280
(RAD54L):inhibitor	20.16	55	1538	411	223822	3.08E-49
(ATXN2):inhibitor	19.95	221	1538	1867	223822	1.24E-186
(MLLT3)[AF4 peptide]:inhibitor	19.54	118	1538	948	223822	3.21E-101
[Peg3 Promoter]:inhibitor	18.08	389	1538	4114	223822	2.93E-302
(MITF):inhibitor	16.78	202	1538	1999	223822	7.99E-158
(PREPL):inhibitor	12.14	153	1538	2018	223822	4.28E-102
(PPP1CA):inhibitor	10.24	164	1538	2578	223822	1.49E-98

* *kA* – the number of Active molecules known to have off target activity

** *kB* – the number of InActive molecules known to have off target activity

*** Molecules that have no experimentally validated targets are not accounted in the table.

### Inhibitors of the human deubiquitinating enzyme (UCH37)

Deubiquitinating enzymes (DUB) represent a group of cysteine proteases that cleave the isopeptide bond between ubiquitin and its conjugated proteins [37]. The HTS assay (PubChem Id 588478) aims to identify small molecule inhibitors of human UCH37, which is tightly associated with the proteasome. Specifically, this screen sought to identify small molecules that inhibit the increase in fluorescence resulting from the UCH37 mediated cleavage of a fluorescent substrate, ubiquitin-7-amido-4-methylcoumarin (Ub-AMC). In total, a library of 329843 small molecules was screened and 1078 molecules were reported as active (efficiently inhibiting UCH37 at low concentration).

The top ten potential off target activities for the molecules inhibiting UCH37 are presented in Table 3. for example, the ability of a molecule to inhibit UCH37 significantly increases the odds (~ 30 fold) for the molecule to inhibit TDP2 (tyrosyl-DNA phosphodiesterase 2). In total, 965 (119 + 846) molecules tested in the assay have been reported in other HTS screens as efficient inhibitors of TDP2 and 119 of them demonstrated potent inhibition of UCH37 in the assay. Observed odds for a molecule to inhibit UCH37 in the screen is ~ 0.005 (2130/238331), while observed odds for the molecules known to inhibit TDP2 is approximately 30 times higher ~ 0.14 (119/846). Thus, we observe strong association between UCH37 and TDP2: molecules targeting one protein have a reasonable chance to target the other as an off target.

Human USPs are considered as novel targets for therapeutic intervention in a number of human cancers, including prostate, colon and breast cancer as well as acute lymphoblastic leukemia [38]. Tyrosyl-DNA phosphodiesterase 2 (TDP2) is implicated in topoisomerase-mediated repair of DNA damage. As a drug target, TDP2 is hypothesized to mediate drug resistance to topoisomerase II inhibition by etoposide [39]. Therefore, inhibition of TDP2 is proposed as a promising approach to overcome intrinsic or acquired resistance to topo II-targeted drug therapy. The observed properties of screened small molecules to inhibit both targets could be exploited for the design of multitarget agents. Considering that the mechanisms of drug action for UCH37 and TDP2 differ significantly, the potential for drugs targeting both of them is likely to be more efficient than targeting independent mechanisms in the cancer cell. Therefore, multi-target drugs affecting several cancer mechanisms represented by UCH37 and TDP2 could be more efficient. On the other hand, TDP2 has been reported to be essential for normal neural function and is required to maintain normal levels of several gene transcripts in the mouse brain during development [40]. Therefore, targeting TDP2 has increased odds to be associated with neurological side effects.

**Table 3:** Top off targets for molecules inhibiting UCH37 (based on data from assay “A screen for small molecule inhibitors of the human deubiquitinating enzyme, UCH37”)

(Target): activity	Odds Ratio	kA*	*KA* (The Number of Active molecules)	kB**	KB*** (The Number of InActive molecules)	*P*-value
(USP1):inhibitor	69.85	158	1074	508	206210	4.73E-211
(PAFAH1B3):inhibitor	55.05	140	1074	560	206210	3.35E-175
(TDP2):inhibitor	30.25	119	1074	846	206210	3.14E-122
(HKDC1):inhibitor	30.11	55	1074	369	206210	5.08E-58
(APAF1):inhibitor	24.98	181	1074	1660	206210	4.65E-169
(WHSC1):inhibitor	21.86	124	1074	1224	206210	2.06E-111
(CASP6):inhibitor	20.33	115	1074	1209	206210	2.07E-100
[RBBP9]:inhibitor	19.4	61	1074	638	206210	1.27E-53
(CTSL1):inhibitor	18.09	98	1074	1138	206210	1.07E-81
[FadD28]:competitors for binfing	17.23	52	1074	607	206210	1.20E-43

* *kA* – the number of Active molecules known to have off target activity

** *kB* – the number of InActive molecules known to have off target activity

*** Molecules that have no experimentally validated targets are not accounted in the table.

### SUMO-lation assays

Targeting SUMOylation has become an important drug discovery trend considering the number of HTS assays developed (see Table 1). Targeting SUMOylation has been considered as a drug discovery strategy to overcome cancer cell resistance to chemo and radiation therapy [41]. In particular, some studies specifically search for small molecule inhibitors of protein-protein interactions mediated by SUMO. The rationale in developing an HTS assay “identification of SUMO1-mediated protein-protein interactions” was a demand for discovery of small molecules that can specifically act as “chemical modulators” of SUMO-mediated signaling. The assay was developed on the principal of time-resolved fluorescence resonance energy transfer (TR-FRET) based on a N-terminal-fluorescein tagged-SUMO 1 specific sequence peptide. The binding event brings the fluorescein acceptor moiety in close proximity to the Tb-donor to allow time-resolved lanthanide fluorescence from the terbium. In total, a library of 362962 small molecules was screened and 1206 molecules were reported as active (efficiently inhibiting SUMO1-mediated protein-protein interactions (PPI) at low concentrations).

The top ten potential off target activities for the small molecule inhibitors of PPI mediated by SUMO are presented in Table 4. for example, the ability of a molecule to inhibit MBD2 significantly increases odds (~ 110 fold) for the molecule to inhibit SUMO1-mediated PPI. In total, 258 (82 + 176) molecules tested in the assay have been reported in other HTS screens as efficient inhibitors of MBD2 and 82 of them demonstrated potent inhibition of SUMO1-mediated PPI. Observed odds for a molecule to inhibit SUMO1-mediated PPI in the screen is ~ 0.0045 (1091/241605), while observed odds for the molecules known to inhibit MBD2 is approximately 100 times higher ~ 0.45 (82/176). Thus, we observe a strong association between MBD2 and SUMO1-mediated PPI: again, molecules targeting one have a reasonable chance to target the other one as an off target.

Among the top potential off targets for the molecules inhibiting SUMO1-mediated PPI we can see proteins commonly associated as drug targets against cancer. For example, MBD2 belongs to the family of methyl-CpG binding proteins [42]. MBD2 mediates epigenetic gene silencing and thus is an attractive target in cancer treatment for reactivation of apoptotic genes as a primary therapeutic mechanism. Another off target WRN is RecQ DNA helicase that participates in suppression of DNA hyper-recombination and repair. WRN was considered as a cancer therapeutic target in hypopharyngeal carcinomas, which have the worst prognosis among head and neck squamous cell carcinomas (HNSCC) with a rapidly rising incidence [43]. It has been shown that WRN is highly expressed in HNSCC, and that siRNA-mediated silencing of the gene suppressed carcinoma cell growth *in vitro*. Therefore, there are several potential off targets for the molecules inhibiting SUMO1-mediated PPI which are potential anticancer targets. As has been already mentioned, this knowledge could be utilized for the development of multitarget drugs and which may therefore provide therapeutic intervention affecting multiple independent anticancer mechanisms.

On the other hand, there are two potential off targets which seem better avoided. The HKDC1 (HexoKinase Domain Containing I) gene encodes mammalian hexokinase. Hexokinases (HKs) catalyse the first step in glucose metabolism and play a major role in regulating the metabolic fate of glucose in the tissues where they are expressed. The dysregulation of glucose is commonly associated with Type 2 diabetes, and activation of HKDC1 is considered as a potential target for diabetes therapeutics while inhibition of HKDC1 could lead to the undesired inhibition of glucose metabolism [44]. Rap guanine nucleotide exchange factor (the gene is known as RAPGEF3, but the protein is usually referred to as EPAC) is closely involved in the regulation of cAMP signaling. EPAC has been implicated in playing important roles in major human pathological conditions such as diabetes and heart disease and EPAC is now considered as a new regulator of cardiac physiopathology [45]. Although its effects are much less well known than the classical cAMP effector, PKA, several studies have investigated the cardiac role of EPAC, providing evidence that EPAC modulates intracellular Ca(2+) levels. In one of the first analyses, it was shown that EPAC can increase the frequency of spontaneous Ca(2+) oscillations in cultured rat cardiomyocytes. Considering that drugs targeting EPAC have increased chances to lead to unexpected cardiac side effects, it would be better to select drug leads with SUMO1-mediated PPI activities that do not possess activity towards RAPGEF3 gene products to minimize these risks.

**Table 4:** Top off targets for molecules inhibiting SUMO1-mediated PPI (based on data from assay “uHTS identification of SUMO1-mediated protein-protein interactions”)

(Target): activity	Odds Ratio	kA*	*KA* (The Number of Active molecules)	kB**	KB*** (The Number of InActive molecules)	*P*-value
(MMP2):inhibitor	144.62	68	1091	111	241605	4.66E-111
(MBD2):inhibitor	111.48	82	1091	176	241605	4.35E-126
(ACP1):inhibitor	105.01	224	1091	593	241605	< 1.57E-280
(PTPN5):inhibitor	88.51	216	1091	672	241605	< 1.57E-280
(BLM):inhibitor	85.02	87	1091	246	241605	3.85E-125
(HKDC1):inhibitor	73.78	125	1091	423	241605	1.17E-171
(WRN):inhibitor	69.34	314	1091	1400	241605	< 1.57E-280
(DNMT1):inhibitor	69.02	459	1091	2516	241605	< 1.57E-280
(RAPGEF3):antagonist	66.59	108	1091	398	241605	9.99E-145

* *kA* – the number of Active molecules known to have off target activity

** *kB* – the number of InActive molecules known to have off target activity

*** Molecules that have no experimentally validated targets are not accounted in the table.

### SENP assays

A group of proteases known as SENPs are involved in both the maturation of SUMO precursors (endopeptidase cleavage) and deconjugation of the targets (isopeptidase cleavage) [46]. Human cells contain seven SENPs (SENPs -1, -2, -3, -5, -6, -7, and -8), and several of these have been characterized as SUMO (or Nedd8) endopeptidases or isopeptidases [47]. The importance of SENPs as drug targets is emphasised by the evidence that SUMOlation regulates cell fate decisions by regulating p53, mdm2, and PML activity [41, 48]. A number of assays covering SENP1, SENP6, SENP7 and SENP8 have been developed recently (see Table 1). The objective of the assays was to identify small molecule inhibitors specific for SENP6, SENP7 and SENP8. The assays utilized an RLRGG-aminoluciferin peptide substrate and used SENP8-dependent deconjugation of the aminoluciferin, which serves as a substrate for the coupled luciferase. In total, libraries covering ~ 300000 small molecules were screened versus the SENP family and several thousand molecules were reported as active (efficiently inhibiting SENP proteases at low concentration). Table 5 reports the top 10 ten potential off target activities for the small molecule inhibitors of SENP6.

**Table 5:** Top off targets for molecules inhibiting SENP6 (based on data from assay “uHTS Luminescent assay for identification of inhibitors of Sentrin-specific protease 6 (SENP6)”)

(Target): activity	Odds Ratio	kA*	*KA* (The Number of Active molecules)	kB**	KB*** (The Number of InActive molecules)	*P*-value
(SENP7):inhibitor	1156.49	4904	5779	1059	219581	< 1.57E-280
(CASP3):inhibitor	1111.03	3950	5779	426	219581	< 1.57E-280
(SENP8):inhibitor	459.86	4734	5779	2142	219581	< 1.57E-280
(PKM):inhibitor	138.06	107	5779	30	219581	1.57E-141
(MMP14):inhibitor transcription	30.53	225	5779	291	219581	1.07E-211
(TNFRSF10B):inhibitor	27.9	1148	5779	1934	219581	< 1.57E-280
(NPC1):activator	27.67	2286	5779	5074	219581	< 1.57E-280
(RAB9A):activator	26.6	2550	5779	6331	219581	< 1.57E-280
(STAT3):inhibitor	23.94	572	5779	1003	219581	< 1.57E-280
(TP53):re-activators of p53 using a Luc reporter	23.47	105	5779	173	219581	2.25E-91

* *kA* – the number of Active molecules known to have off target activity

** *kB* – the number of InActive molecules known to have off target activity

*** Molecules that have no experimentally validated targets are not accounted in the table.

The ability of a molecule to inhibit SENP6 observed in the assay was very strongly correlated with the ability of the molecule to inhibit other SENP family members: SENP7 and SENP8. This could be partially explained by technical bias. The assays are based on Caspase-3 dependent deconjugation of the aminoluciferin, which serves as a substrate for the coupled Ultra-GloTM luciferase. Therefore, molecules inhibiting CASP3 will also provide an artefactual signal in the assay. For this reason several control assays have been carried out in order to deprioritize compounds that inhibit Caspase-3. As it can be seen from Table 5, the majority of molecules detected in the initial screen for potential SENP inhibitors are also Caspase-3 inhibitors. Considering the numbers, we can see that more than 80 percent of active molecules are non-specific in relation to either CASP3 or other family members.

As in previous cases, there a number of off target activities which can be utilized for development of multitarget drugs as well as some specific off targets which are better to be avoided. For example, the molecules inhibiting the SENP family seem to have increased odds (~23 fold) to reactivate p53. Lack of p53 expression or expression of mutant p53 is common in human cancers and is associated with increased tumor growth and resistance to therapies [49, 50]. Significant efforts toward pharmaceutical reactivation of defective p53 by small molecules are considered as an independent anticancer therapeutic strategy [51-54]. Indeed, reactivated p53 can lead to tumor destruction [55]. Therefore, selecting drug candidates for the next developmental stages from SENPs screens where reactivation of p53 is a potential off target activity would increase the chances of developing potent anticancer agents that target independent anticancer mechanisms.

On the other hand, those molecules inhibiting the SENP family seem to have increased odds (~140 fold) to inhibit PKM (pyruvate kinase, muscle). Pyruvate kinase is a key glycolytic enzyme which is expressed as several isoforms in many cell types (liver, pancreatic cells, small intestine and so on). PKM has been reported to have important roles in many diseases including cancer and PKM polymorphisms have been shown to be associated with type 2 diabetes [56]. Considering the complicated and controversial role of PKM in human diseases, it would be better to avoid potential drug leads that additionally target PKM.

## DISCUSSION

There have been significant efforts in recent years to discover small molecular potent inhibitors of ubiquitin–proteasome or ubiquitin-like signalling systems including a number of high-throughput screening projects which reported thousands of potential drug lead molecules efficiently targeting disease specific UPS-UBL components *in vitro* [18, 20-22]. A critical step in drug discovery is a selection of those drug leads that have better chances of success in clinical trials [57]. Selected molecules are subjected to a number of preclinical tests to address potential issues with pharmacodynamics, pharmacokinetics, ADME properties and potential toxicity. Considering the low rate of success in current drug discovery, any rationale for improving the selection process of drug leads that increases the chances of avoiding further safety [58] or efficacy issues [59-61] is of paramount importance.

It is now commonly accepted that polypharmacology (the ability to affect multiple independent protein targets) is a basic property of small molecules [60, 62-64]. Therefore, understanding the potential off target spectrum of molecules targeting UPS(UBL) is of practical importance [65, 66]. Here we have explored the abundance of publicly available high-throughput screening assay data in order to understand polypharmacology of small molecules tested in UPS-UBL high-throughput screens (Table 1). To do this, we interrogated (> 1000) HTS target oriented screens available in the public domain (PubChem repository [25]), which cover a wide spectrum of commonly accepted drug targets for various diseases. The opportunity of such statistical analysis stems from the fact that the chemical libraries used in most of these high-throughput screens share a significant number of common molecules. Thus we computed for each UPS(UBL) target the off target spectrum by counting the number of molecules which are reported to be active/inactive between a pair of assays (UPS-UBL assay versus off target assay) and thus computed the shift in odds for a molecule to target a specific component of the UPS while being experimentally validated to target another given protein. For each UPS(UBL) target we derived the list of top off targets (off target spectrum) which have high odds to be targeted by potential UPS(UBL) drug leads.

Polypharmacology of a small molecule could be either beneficial (co-targeting of certain proteins could increase efficiency) or the opposite (co-targeting of certain proteins could lead to undesired side effects). Here we have demonstrated that information for potential off targets for molecules targeting different UPS components could be utilized to design multi-target drugs, which are able to affect independent disease specific mechanisms. For example, from the HTS data we observed a strong correlation between drugs targeting Ubc13 and APAF1. Both targets could be potentially implicated in treatment of the pathogenic aggregation of mutant proteins in neurodegenerative diseases such as Huntingtons disease, although targeting different mechanisms. This observation (pharmacological association between Ubc13 and APAF1) can be utilized in future efforts to identify potent drug leads that inhibit both targets and, therefore, by affecting two independent mechanisms in neurodegenerative diseases the potential efficacy of the multitarget therapy is enhanced.

Finally, we have demonstrated that information for potential off targets for molecules targeting different UPS components could be utilized to reduce risks associated with potential side effects of drug leads. Among pharmacologically associated off targets for different UPS targets there are multiple proteins known to be associated with various disease conditions such as cardiovascular or diabetes. For design of anticancer agents, for example, these types of off targets should be avoided as inhibition or activation of these proteins might have undesirable corresponding side effects.
